# Sex-Specific Associations Between Sarcopenia and Obesity Parameters and Falls in Korean Older Adults: A Cross-Sectional Analysis of the Korean Frailty and Aging Cohort Study

**DOI:** 10.3390/medicina62061101

**Published:** 2026-06-05

**Authors:** Yunjung Rho, Seongmin Choi, Miji Kim, Yunsoo Soh, Chang Won Won

**Affiliations:** 1 Department of Physical Medicine and Rehabilitation Medicine, Kyung Hee University Medical Center, Seoul 02447, Republic of Korea; allyrho@naver.com (Y.R.); ysisminee@naver.com (S.C.); 2 Department of Health Sciences and Technology, College of Medicine, Kyung Hee University, Seoul 02447, Republic of Korea; mijiak@khu.ac.kr; 3 Department of Family Medicine, Kyung Hee University Medical Center, Seoul 02447, Republic of Korea

**Keywords:** aging, sarcopenia, obesity, central obesity, sarcopenic obesity, Asian Working Group for Sarcopenia, falls

## Abstract

*Background and Objectives*: Aging is associated with sarcopenia and increased adiposity, which may impair mobility and increase fall risk. Although sarcopenic obesity is associated with an increased fall risk compared with either condition alone, evidence regarding sex-specific associations remains limited. This study aimed to examine the sex-specific associations between sarcopenia, obesity parameters, and falls among older Korean community-dwellers. *Materials and Methods*: This cross-sectional study analyzed baseline data from the Korean Frailty and Aging Cohort Study, including 2039 participants aged 70–84 years (men, 973; women, 1066). Sarcopenia was defined using the Asian Working Group for Sarcopenia 2025 criteria, and obesity was categorized as general (body mass index and percentage body fat [PBF]) or central obesity (waist circumference [WC] and conicity index [C-index]). Falls were assessed by self-report. Logistic regression analyses were performed after adjusting for potential confounders. *Results:* Among 2039 participants, 395 (19.4%) reported falls. In the total population, higher PBF and C-index were independently associated with increased fall risk after adjustment. Men showed significantly higher odds of falls with low handgrip strength, high WC, PBF, and C-index than women. In women, only low appendicular skeletal muscle mass index was independently associated with falls. Sarcopenic obesity was not significantly associated with falls in either sex. *Conclusions:* Although sarcopenic obesity itself was not independently associated with falls in either sex, distinct sex-specific associations were observed between individual components of sarcopenia, obesity, and fall risk among older Korean adults. Reduced muscle strength and central obesity were more strongly associated with falls in men, whereas reduced muscle mass was independently associated with falls in women. These findings suggest that sex-specific approaches targeting muscle function and body composition may be important for fall prevention in aging populations.

## 1. Introduction

Aging induces profound alterations in body composition characterized by progressive skeletal muscle loss (sarcopenia), increased adiposity, visceral fat redistribution, and intramuscular lipid infiltration [1]. The coexistence of sarcopenia and obesity can impair mobility through multiple mechanisms: reduced muscle quality limits force generation; excess fat increases the biomechanical load; and central obesity shifts the center of mass, thereby affecting postural stability and proprioception [2]. Given their clinical importance, the definitions of sarcopenia and sarcopenic obesity have recently been revised and consolidated [2,3]. The updated Asian Working Group for Sarcopenia (AWGS) consensus defines sarcopenia as the coexistence of low skeletal muscle mass and strength. Physical performance measures were excluded from the diagnostic criteria and categorized as outcome measures [3]. Sarcopenic obesity, defined as the coexistence of sarcopenia and obesity, is a particularly deleterious geriatric syndrome [4]. This condition is associated with accelerated functional decline, reduced quality of life, metabolic comorbidities (including diabetes and cardiovascular disease), and increased all-cause mortality [5]. The European Society for Clinical Nutrition and Metabolism and the European Association for the Study of Obesity consensus recommends a three-step approach for diagnosis, including screening based on obesity measures (body mass index [BMI] or waist circumference [WC]) and suspected muscle dysfunction, diagnostic confirmation using body composition and muscle function assessments, and staging based on sarcopenic obesity-related complications [2]. These age-related changes exhibit notable sex-specific patterns: total fat mass increases predominantly in women, whereas percent body fat (PBF) increases in both sexes. Visceral adipose tissue accumulates after 40 years of age because of hormonal changes, such as declining testosterone levels in men and postmenopausal estrogen deficiency in women [6]. Morphologically, excess abdominal fat transforms body shape from cylindrical to a characteristic “double-cone” appearance, which is quantifiable through the conicity index (C-index)—a geometric measure integrating WC, height, and weight capturing central obesity more sensitively than BMI alone [7]. Notably, previous studies have indicated that measures of abdominal adiposity, such as WC, are more strongly associated with chronic diseases and fall risk than BMI [8,9].

Falls are the leading cause of injury-related morbidity among older community-dwellers, generating substantial healthcare costs and precipitating fear of falling, activity restrictions, and institutionalization [10]. Identifying modifiable risk factors for falls remains a critical public health priority. Prior analysis using Korean Frailty and Aging Cohort Study (KFACS) data identified sex-specific associations between sarcopenia and falls; low muscle strength and physical performance in men and low muscle mass in women were associated with falls [11]. However, sarcopenia itself was not associated with falls in either sex, highlighting that sarcopenia alone insufficiently explains the fall etiology. The additive burden of obesity likely amplifies risk through distinct biomechanical and metabolic pathways beyond sarcopenia alone [12]. Emerging international evidence links sarcopenic obesity to increased fall risk. Among 10,905 Chinese adults aged ≥ 60 years, sarcopenic obesity showed a significantly increased fall risk in a 5-year longitudinal analysis, whereas sarcopenia alone did not demonstrate a statistically significant association with falls [13]. These findings suggest that the coexistence of obesity and sarcopenia confers a greater risk of falls than sarcopenia alone in older adults. A systematic review and meta-analysis of 26 studies with 37,124 older adults found that sarcopenic obesity significantly increased fall risk by 30% compared with healthy controls [14].

To our knowledge, no study has comprehensively examined the sex-specific associations between sarcopenia, obesity parameters, and falls in older Korean adults. Therefore, this study aimed to investigate the association of recently revised sarcopenia parameters and various obesity parameters with falls, stratified by sex, among older Korean community-dwellers.

## 2. Materials and Methods

### 2.1. Study Population

Our study used data from the KFACS, a multicenter longitudinal study with a baseline survey conducted between 2016 and 2017 [15]. Among 3011 older community-dwellers aged 70–84 years, 2401 underwent dual-energy X-ray absorptiometry (DEXA) using Hologic (Marlborough, MA, USA) or GE Healthcare Lunar (Chicago, IL, USA) systems at eight university hospital centers. To ensure methodological homogeneity, participants whose body composition was assessed using bioelectrical impedance analysis (*n* = 610) were excluded.

**Figure 1:**
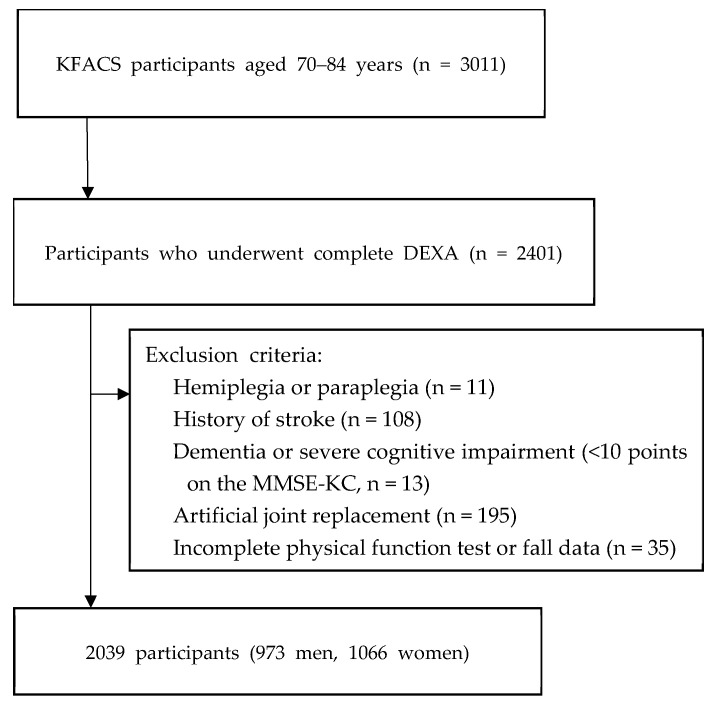
Flowchart of the participant recruitment process. KFACS, Korean Frailty and Aging Cohort Study; DEXA, dual-energy X-ray absorptiometry; MMSE-KC, Mini-Mental Status Examination in the Korean version of the CERAD assessment packet.

Participants with hemiplegia or paraplegia (*n* = 11), a history of stroke (*n* = 108), diagnosis of dementia, Mini-Mental Status Examination—Korean version (MMSE-KC) score < 10 (*n* = 13), history of artificial joint replacement (*n* = 195), and incomplete data on physical function tests or falls (*n* = 35) were excluded (Figure 1). The Korean Frailty and Aging Cohort Study (KFACS) protocol was approved by the Institutional Review Board of the Clinical Research Ethics Committee of Kyung-Hee University Medical Center (IRB No. 2015-12-103). This study was conducted in accordance with the ethical standards of the Declaration of Helsinki. Written informed consent was obtained from all participants.

### 2.2. Definitions

#### 2.2.1. Sarcopenia

Our study used the term “sarcopenia” as defined by the AWGS consensus, updated in 2025 [3]. The AWGS developed diagnostic criteria for sarcopenia from 2014 to 2019, and the 2025 version was recently released. According to AWGS 2025 [3], participants with concurrent low skeletal muscle mass and low muscle strength were defined as the sarcopenia group. Physical performance measures were excluded from the diagnostic criteria and instead categorized as outcome measures [3]. Skeletal muscle mass was assessed using the appendicular skeletal muscle mass index (ASMI), with low muscle mass defined as ASMI < 7.0 kg/m^2^ in men and < 5.4 kg/m^2^ in women. Muscle strength was assessed using handgrip strength (HGS), with low muscle strength defined as HGS < 28 kg in men and <18 kg in women. Sarcopenia was defined when both criteria were met.

#### 2.2.2. General Obesity and Central Obesity

According to the World Health Organization (WHO), overweight and obesity are defined as abnormal or excessive fat accumulation that presents health risks [16]. Although BMI is the most commonly used measure of obesity, central obesity is a better surrogate for visceral adiposity and is strongly associated with falls and multiple obesity-related disorders [12,17]. Nevertheless, BMI remains a widely used and clinically accessible indicator in epidemiological and sarcopenic obesity research. Therefore, both general and central obesity parameters were included in the analysis [9]. General obesity parameters were assessed using BMI and PBF, whereas central obesity parameters were evaluated using WC and the C-index [7,9].

BMI was calculated as weight in kilograms divided by height in meters squared (kg/m^2^). Considering that obesity thresholds are lower in Asian populations, a BMI ≥ 25 kg/m^2^ was used to define obesity with an increased risk of comorbidities based on the WHO Asia-Pacific guidelines [18].

PBF was calculated as fat mass divided by total body mass multiplied by 100 using DEXA data. Obesity was defined using PBF cutoff values of ≥25% for men and ≥35% for women [19].

WC was assessed at the midpoint between the iliac crest and the lower edge of the 12th rib, with cutoff points of ≥90 cm for men and ≥85 cm for women [20].

The C-index, developed by Valdez [7], is calculated using weight, height, and WC to identify abdominal obesity:

$$C-index=\frac{WC(m)}{0.109\times \sqrt{\frac{body\;weight(kg)}{height(m)}}}.$$

This index describes how abdominal fat accumulation changes the body from a cylindrical shape to a double-cone shape. Based on previous research, the C-index was considered elevated at values ≥ 1.25 for men and ≥1.18 for women [21]. Both waist circumference (WC) and the conicity index (C-index) were included to capture complementary aspects of central adiposity.

#### 2.2.3. Falls

Falls were assessed using self-reported responses to the question “Have you experienced any falls in the past year?”, with response options of yes, no, or unknown. Participants who responded with “unknown” were excluded from the analysis.

#### 2.2.4. Sarcopenic Obesity

Sarcopenic obesity, defined as the presence of sarcopenia in conjunction with either a high BMI or increased WC [2]. Sarcopenic obesity (SO) was assessed using an integrated approach combining the ESPEN/EASO consensus framework and the AWGS 2025 [3] criteria for sarcopenia. Obesity-related parameters were defined according to the WHO Asia-Pacific guidelines. This approach was used to improve population-specific applicability in older Korean adults.

### 2.3. Other Measurements

Trained investigators collected the demographic data, medical status, and body composition analyses used in this study. Current smokers were defined as individuals who were currently smoking, regardless of cigarette consumption. Alcohol drinkers were defined as participants who consumed alcohol at least once a week.

### 2.4. Statistical Analyses

Continuous variables were compared using the *t*-test, and categorical variables were compared using Pearson’s chi-square test. Given the large sample size, parametric tests were considered appropriate for the analysis of continuous variables. Fisher’s exact test was used when the expected frequency in any cell was <5. Continuous variables are reported as mean ± standard deviation, and categorical variables as number (ratio, %). The unadjusted and fully adjusted models were derived from logistic regression analyses. To minimize potential multicollinearity among adiposity indicators, BMI, PBF, WC, and C-index were entered into separate logistic regression models. The results are presented as odds ratios (ORs) and 95% confidence intervals (CIs), rounded to two decimal places. The fully adjusted model included age, marital status, osteoporosis, income, education level, and MMSE-KC score as potential confounders. These variables were selected based on both clinical relevance and baseline differences between fallers and non-fallers, as cognitive status and social determinants have been reported to influence fall risk in older adults [22,23]. All data in the study were analyzed using IBM SPSS Statistics for Windows, version 25.0 (IBM Corp., Armonk, NY, USA), and *p*-values < 0.05 were considered statistically significant. No adjustment for multiple comparisons was applied due to the exploratory nature of the analyses.

## 3. Results

Table 1 presents the baseline characteristics of the study population according to sex. Among the 2039 participants, 973 were men, and 1066 were women. Women showed higher mean BMI (24.6 ± 3.0 vs. 23.9 ± 2.9 kg/m^2^) and PBF (37.0 ± 6.0 vs. 26.6 ± 6.1%), as well as a higher prevalence of BMI-defined obesity (42.5% vs. 34.3%), WC-defined central obesity (58.5% vs. 44.4%), PBF-defined obesity (68.5% vs. 63.9%), and C-index-defined central obesity (93.4% vs. 75.7%) than men. In contrast, men showed a higher mean WC (88.6 ± 8.5 vs. 86.4 ± 8.5 cm) than women. Sarcopenia-related characteristics, such as HGS (32.4 ± 5.7 vs. 21.2 ± 3.9 kg) and ASMI (7.0 ± 0.8 vs. 5.8 ± 0.7 kg/m^2^), were higher in men. Additionally, the prevalence of sarcopenia and sarcopenic obesity was higher in men than in women.

The baseline characteristics stratified by fall history are presented in Table 2. A total of 395 participants reported falls, whereas 1644 did not. Fallers differed significantly from non-fallers in several socioeconomic and clinical factors, including marital status, monthly income, education level, MMSE-KC score, and osteoporosis. Furthermore, fallers had significantly higher mean PBF (33.6 ± 7.8 vs. 31.6 ± 8.0%, *p* < 0.01) and C-index (1.3 ± 0.1 vs. 1.3 ± 0.1, *p* = 0.001), as well as a higher prevalence of WC-defined central obesity (57.5% vs. 50.4%, *p* = 0.012), PBF-defined general obesity (42.8% vs. 33.7%, *p* < 0.01), and C-index-defined central obesity (71.6% vs. 65.0%, *p* = 0.012) than non-fallers. Conversely, sarcopenia-related measures, including HGS (24.4 ± 6.5 vs. 27.1 ± 7.5 kg, *p* < 0.01) and ASMI (6.2 ± 1.0 vs. 6.5 ± 1.0 kg/m^2^, *p* < 0.01), were lower in fallers than in non-fallers.

Table 3 compares the sarcopenia and obesity parameters between the non-faller and faller groups according to sex. Among men, fallers exhibited significantly lower HGS (30.5 ± 5.5 vs. 32.8 ± 5.7 kg, *p* < 0.01) and ASMI (6.9 ± 0.8 vs. 7.1 ± 0.8 kg/m^2^, *p* = 0.047) than non-fallers. Additionally, male fallers had a higher PBF (27.6 ± 6.6 vs. 26.4 ± 6.0%, *p* = 0.036) and C-index (1.3 ± 0.1 vs. 1.3 ± 0.1, *p* = 0.013) and a significantly higher prevalence of sarcopenia (21.5% vs. 13.9%, *p* = 0.018). In contrast, among women, the only significant difference was a higher C-index in the faller group (1.3 ± 0.1 vs. 1.3 ± 0.1, *p* = 0.032), whereas other parameters did not differ significantly. The prevalence of sarcopenic obesity did not differ significantly between fallers and non-fallers of either sex.

Table 4 presents the results of the logistic regression analysis examining the associations between the components of sarcopenia and obesity and the risk of falls. As shown in Table 4, fallers in the total group showed significantly higher odds of high PBF, WC, and C-index in the unadjusted model. After adjustment for potential confounders, high PBF (OR, 1.46; 95% CI, 1.14–1.88) and high C-index (OR, 1.52; 95% CI, 1.06–2.18) remained independently associated with an increased risk of falls in the total group. When stratified by sex, male fallers showed significant associations with low HGS, high PBF, high WC, and sarcopenia in the unadjusted model. After adjustment, male faller group showed statistically significant higher odds of low HGS (OR, 1.57; 95% CI, 1.02–2.42), high WC (OR, 1.54; 95% CI, 1.07–2.21), high PBF (OR, 1.90; 95% CI, 1.26–2.86), and high C-index (OR, 1.59; 95% CI, 1.00–2.53). Conversely, for women, only low ASMI (OR, 1.41; 95% CI, 1.03–1.93) showed a significant independent association with a history of fall in the fully adjusted model. An association between sarcopenic obesity and falls was observed in both the total (OR, 1.02; 95% CI, 0.60–1.73) and male (OR, 1.30; 95% CI, 0.66–2.58) groups, but these did not reach statistical significance.

Additional analyses were conducted to explore the associations of sarcopenia and obesity parameters with more severe clinical outcomes, specifically multiple falls (defined as ≥2 falls within the past year) and fall-related fractures. In additional analyses, low HGS was associated with multiple falls in the unadjusted total population (OR, 1.59; 95% CI, 1.08–2.35), whereas low HGS (OR, 3.58; 95% CI, 1.47–8.72) and sarcopenia (OR, 3.15; 95% CI, 1.24–8.04) were associated with fall-related fractures only in men. However, these associations were not significant in the fully adjusted model. No obesity-related parameters were independently associated with multiple falls or fractures, suggesting a limited predictive power for severe outcomes in this cohort.

## 4. Discussion

This study examined the sex-specific relationships among sarcopenia, obesity parameters, and fall risk among older Korean community-dwellers. These results indicated that these associations differed according to sex. Among men, low HGS was significantly associated with falls, whereas among women, low ASMI was significantly associated with falls. However, in the overall group, no significant association was observed between sarcopenia and falls. This finding is consistent with that of a previous study [11]. In men, declines in muscle strength and physical performance are more closely linked to functional impairment and fall risk, whereas in women, body composition, including reduced muscle mass and increased fat mass, may have a greater impact on balance and fall risk [11,24]. Regarding obesity parameters, high PBF and high C-index were significantly associated with falls in the total population after adjusting for covariates. In men, high PBF, WC, and C-index were associated with higher odds of falls, even after full adjustment. Conversely, in women, none of the obesity parameters were significantly associated with falls.

Although both are general obesity parameters, PBF showed a significant association with falls in both the total and male groups, whereas BMI was not associated with falls. This is because BMI reflects only body weight relative to height and does not distinguish between muscle mass, fat mass, or fat distribution. In contrast, PBF reflects the proportion of fat relative to total body weight and, therefore, directly affects balance, muscle strength, and mobility [25,26]. Consequently, in older adults, in whom fat mass increases and muscle mass decreases, BMI may fail to identify sarcopenic obesity. Moreover, central obesity parameters, such as WC and C-index, independently predicted falls in men, suggesting that central obesity plays a critical role in male fall risk. Unlike general obesity, central obesity reflects abdominal fat; increased abdominal fat shifts the center of mass forward, impairs balance and gait stability, and reduces physical performance, thereby more directly increasing the risk of falls [9,27]. Previous studies have not demonstrated a direct association between the C-index, which was used in this study as an indicator of central obesity, and falls. However, higher C-index values have been associated with frailty and cognitive decline [28,29], which are well-established risk factors for falls [22,30], suggesting a potential association with fall risk. Furthermore, sex-specific differences observed in the association between obesity parameters and falls may be explained by differences in fat distribution, activity, and environmental factors between men and women [23,31]. Men tend to accumulate more central and visceral fat, whereas women preferentially store subcutaneous fat in the gluteofemoral region, including the hips and thighs [31]. Consequently, increased abdominal fat, which is more prevalent in men, leads to an anterior shift in the center of mass, thereby compromising balance control and gait stability [9,32,33]. In contrast, fall risk in women has been reported to be influenced not only by changes in body composition but also by environmental factors, medication use, and visual function [23,34].

Although sarcopenic obesity was not significantly associated with falls in our study, previous studies have suggested potential mechanisms linking sarcopenic obesity to impaired balance and mobility [35]. The coexistence of reduced muscle function and excess adiposity may negatively affect gait stability, postural control, and physical performance. [35,36]. However, our findings suggest that the individual components of sarcopenia and obesity, rather than the combined sarcopenic obesity phenotype itself, may have distinct sex-specific associations with fall risk in this population. The lack of a significant association may also reflect differences in study populations, diagnostic criteria, and the relatively healthy characteristics of the KFACS cohort.

Our findings have important clinical implications for fall prevention strategies in older adults. Screening for sarcopenia and obesity should be integrated into comprehensive geriatric assessments with particular attention to sex-specific risk patterns. Men with low muscle strength and central obesity require early interventions to prevent falls, whereas women with low muscle mass require careful attention. Notably, central obesity alone, as measured by WC and C-index, was significantly associated with falls in men, highlighting the importance of managing abdominal obesity. Interventions should target sex-specific impairments in muscle function and body composition in older adults by increasing muscle mass and strength while reducing excess fat mass, particularly visceral adiposity, through multimodal approaches combining resistance exercise, nutritional optimization with adequate protein intake, and caloric restriction.

Although a previous study using KFACS data from a single baseline year reported that higher PBF was associated with an increased risk of fall-related fractures [9], we did not find a significant association between obesity-related parameters and such fractures in the present study. This inconsistency may partly stem from differences in the analytical samples, as our study included participants from multiple baseline years, resulting in a larger and more heterogeneous cohort. Furthermore, the lack of significant associations may be partly explained by the relatively low event rate of severe outcomes, such as fall-related fractures or multiple falls, within the KFACS cohort. This limited number of events likely reduced the statistical power required to identify independent associations between these specific outcomes. Additionally, variations in sample size, inclusion criteria, and modeling approaches, especially in the selection of covariates, could have contributed to the differing results. Notably, fall-related fractures are relatively rare and influenced by multiple factors, including bone quality, fall mechanics, and comorbid conditions. Further longitudinal studies with larger sample sizes are warranted to clarify the complex relationships between sarcopenia, obesity-related parameters, and severe fall-related outcomes.

This study had several limitations. First, the cross-sectional design precluded causal inferences regarding the relationships between sarcopenia, obesity parameters, and falls. Longitudinal studies are required to establish temporal relationships. Second, fall history was assessed through self-reporting, which may have been subject to recall bias. In addition, falls were assessed using a binary yes/no question, and participants with uncertain responses were excluded, which may have introduced misclassification or selection bias and affected the observed associations. Moreover, objective functional measures related to fall risk, such as balance or gait performance assessments, were not included and may have provided further insight into mobility impairments associated with falls. Third, the study population was limited to older Korean community-dwellers aged 70–84 years, which may limit the generalizability to other populations or age groups. In addition, the exclusion criteria and community-based recruitment may have resulted in a relatively healthier and more functional study population compared with the general older population. Fourth, the cutoff values for the obesity indices were determined based on previous studies [18,19,20,21,37]; thus, future research should consider adopting age- and ethnicity-specific thresholds rather than applying uniform criteria. Fifth, the use of two different dual-energy X-ray absorptiometry (DEXA) systems (Hologic and GE Lunar) across multiple centers without formal cross-calibration may have introduced measurement variability. Sixth, several potential confounders, including medication use, physical activity, and visual function, were not available for adjustment and may have influenced the observed associations. Finally, although previous studies suggested a link between sarcopenic obesity and falls [12,13], our study did not find a statistically significant association. This may be due to the relatively healthy nature of the KFACS cohort, resulting in a small sample size of participants with sarcopenic obesity, as well as heterogeneity in sarcopenic obesity definitions across studies, highlighting the need for further research.

## 5. Conclusions

This study demonstrated that specific components of sarcopenia and obesity are significantly associated with falls in older Korean adults, with distinct sex-specific patterns. Men with low muscle strength and central obesity showed an increased risk of falls, whereas women with low muscle mass showed an increased risk of falls. These findings underscore the importance of sex-specific approaches to sarcopenia, obesity assessment, and interventions in fall prevention programs. Early identification and management of sarcopenic obesity through multimodal interventions targeting both muscle preservation and optimal body composition may reduce fall risk and improve the quality of life in aging populations. Further studies that apply ethnicity- and age-specific definitions are warranted to elucidate the relationship between sarcopenic obesity and falls in Asian populations, particularly among older Korean adults.

## Figures and Tables

**Table 1:** Baseline characteristics of the participants according to sex.

Characteristic	Men (*n* = 973)	Women (*n* = 1066)
Demographic characteristics		
Age (years)	76.4 ± 3.9	75.5 ± 3.9
Height (cm)	165.0 ± 5.7	151.9 ± 5.3
Weight (kg)	65.3 ± 9.0	56.9 ± 8.0
Obesity-related parameters		
BMI (kg/m^2^)	23.9 ± 2.9	24.6 ± 3.0
Obesity by BMI (≥25 kg/m^2^)	334 (34.3)	453 (42.5)
Waist circumference (cm)	88.6 ± 8.5	86.4 ± 8.5
Central obesity by WC (men ≥ 90 cm, women ≥ 85 cm)	432 (44.4)	624 (58.5)
PBF	26.6 ± 6.1	37.0 ± 6.0
Obesity by PBF (men ≥ 25%, women ≥ 35%)	622 (63.9)	730 (68.5)
C-index	1.3 ± 0.1	1.3 ± 0.1
Central obesity by C-index (men ≥ 1.25, women ≥ 1.18)	737 (75.7)	996 (93.4)
Socioeconomic and clinical characteristics		
Marital status, married	881 (90.5)	512 (48.0)
Residency (%)		
Urban	760 (78.1)	885 (83.0)
Rural	209 (21.5)	172 (16.1)
Income per month		
>3 million Korean won	255 (26.2)	144 (13.5)
1–3 million Korean won	370 (38.0)	359 (33.7)
<1 million Korean won	309 (31.8)	482 (45.2)
Education level		
No formal education	67 (6.9)	284 (26.6)
Elementary/middle/high school	592 (60.8)	668 (62.7)
College/university	313 (32.2)	113 (10.6)
Hypertension	514 (52.8)	616 (57.8)
Diabetes mellitus	230 (23.6)	195 (18.3)
Dyslipidemia	233 (23.9)	419 (39.3)
Osteoporosis	26 (2.7)	267 (25.0)
Depression	17 (1.7)	33 (3.1)
Alcohol drinking	331 (34.0)	40 (3.8)
Current smoker	108 (11.1)	11 (1.0)
MMSE-KC	26.5 ± 2.7	25.3 ± 3.4
Sarcopenia-related parameters		
Skeletal muscle function		
HGS (kg)	32.4 ± 5.7	21.2 ± 3.9
Body composition		
ASMI (kg/m^2^)	7.0 ± 0.8	5.8 ± 0.7
Sarcopenia ^a^	146 (15.0)	82 (7.7)
Sarcopenic obesity ^b^	60 (6.2)	33 (3.1)

Values are means ± standard deviation or n (%). BMI, body mass index; WC, waist circumference; PBF, percentage body fat; C-index, conicity index; MMSE-KC, Mini-Mental Status Examination in the Korean version of the CERAD assessment packet; HGS, hand grip strength; ASMI, Appendicular Skeletal Muscle Mass Index. ^a^ Sarcopenia: low ASMI (<7.0 kg/m^2^ for men and <5.4 kg/m^2^ for women) and low HGS (<28 kg for men and <18 kg for women). ^b^ Sarcopenic obesity: sarcopenia with either high BMI (≥25 kg/m^2^) or high WC (≥90 cm for men and ≥85 cm for women).

**Table 2:** Baseline characteristics of the participants according to fall history.

Characteristic	Faller (*n* = 395)	Non-Faller (*n* = 1644)	*p*
Demographic Characteristics			
Age (years)	76.3 ± 3.8	75.8 ± 4.0	0.029 *
Height (cm)	156.2 ± 8.6	158.6 ± 8.5	<0.01 **
Weight (kg)	59.8 ± 9.6	61.1 ± 9.4	0.015 *
Obesity-Related Parameters			
BMI (kg/m^2^)	24.5 ± 3.1	24.3 ± 2.9	0.166
Obesity by BMI (≥25 kg/m^2^)	159 (40.3)	628 (38.2)	0.452
Waist circumference (cm)	88.0 ± 8.8	87.4 ± 8.5	0.175
Central obesity by WC (men ≥ 90 cm, women ≥ 85 cm)	227 (57.5)	829 (50.4)	0.012 *
PBF	33.6 ± 7.8	31.6 ± 8.0	<0.01 **
Obesity by PBF (men ≥ 25%, women ≥ 35%)	283 (71.6)	1069 (65.0)	0.012 *
Conicity index	1.3 ± 0.1	1.3 ± 0.1	0.001 *
Central obesity by C-index (men ≥ 1.25, women ≥ 1.18)	356 (90.1)	1377 (83.8)	0.001 *
Socioeconomic and Clinical Characteristics			
Marital status, married	230 (58.2)	1163 (70.7)	<0.01 **
Residency (%)			
Urban	323 (81.8)	1322 (80.4)	0.799
Rural	70 (17.7)	311 (18.9)	
Income per month			
>3 million Korean won	64 (16.2)	335 (20.4)	0.001 *
1–3 million Korean won	120 (30.4)	609 (37.0)	
<1 million Korean won	180 (45.6)	611 (37.2)	
Education level			
No formal education	93 (23.5)	258 (15.7)	0.001 *
Elementary/middle/high school	236 (59.7)	1024 (62.3)	
College/university	65 (16.5)	361 (22.0)	
Hypertension	227 (57.5)	903 (54.9)	0.514
Diabetes mellitus	93 (23.5)	332 (20.2)	0.243
Dyslipidemia	137 (34.7)	515 (31.3)	0.37
Osteoporosis	83 (21.0)	210 (12.8)	<0.01 **
Depression	16 (4.1)	34 (2.1)	0.063
Alcohol drinking	64 (16.2)	307 (18.7)	0.315
Current smoker	21 (5.3)	98 (6.0)	0.581
MMSE-KC	25.3 ± 3.3	26.0 ± 3.0	0.001
Sarcopenia-Related Parameters			
Skeletal muscle function			
HGS (kg)	24.4 ± 6.5	27.1 ± 7.5	<0.01 **
Body composition			
ASMI (kg/m^2^)	6.2 ± 1.0	6.5 ± 1.0	<0.01 **
Sarcopenia ^a^	50 (12.7)	178 (10.8)	0.300
Sarcopenic obesity ^b^	19 (4.8)	74 (4.5)	0.792

Values are means ± standard deviation or n (%). BMI, body mass index; WC, waist circumference; PBF, percentage body fat; C-index, conicity index; MMSE-KC, Mini-Mental State Examination in the Korean version of the CERAD assessment packet; HGS, hand grip strength; ASMI, Appendicular Skeletal Muscle Mass Index. ^a^ Sarcopenia: low ASMI (<7.0 kg/m^2^ for men and <5.4 kg/m^2^ for women) and low HGS (<28 kg for men and <18 kg for women). ^b^ Sarcopenic obesity: sarcopenia with either high BMI (≥25 kg/m^2^) or high WC (≥90 cm for men and ≥85 cm for women). Statistical significance: * p < 0.05 and ** *p* < 0.01.

**Table 3:** Sarcopenia and obesity parameters of the faller and non-faller group according to sex.

Characteristic	Men		*p*	Women		*p*
	Fall (*n* = 144)	Non-Fall (*n* = 829)		Fall (*n* = 251)	Non-Fall (*n* = 815)	
HGS (kg)	30.5 ± 5.5	32.8 ± 5.7	<0.01 **	20.9 ± 3.8	21.3 ± 3.9	0.164
ASMI (kg/m^2^)	6.9 ± 0.8	7.1 ± 0.8	0.047*	5.8 ± 0.8	5.8 ± 0.7	0.11
BMI (kg/m^2^)	24.1 ± 2.9	23.9 ± 2.9	0.624	24.7 ± 3.2	24.6 ± 3.0	0.512
PBF (%)	27.6 ± 6.6	26.4 ± 6.0	0.036 *	37.1 ± 6.1	36.9 ± 6.0	0.682
WC (cm)	89.6 ± 8.4	88.4 ± 8.5	0.130	87.1 ± 8.9	86.2 ± 8.4	0.169
C-index	1.3 ± 0.1	1.3 ± 0.1	0.013 *	1.3 ± 0.1	1.3 ± 0.1	0.032 *
Sarcopenia ^a^	31 (21.5)	115 (13.9)	0.018 *	19 (7.6)	63 (7.7)	0.934
Sarcopenic obesity ^b^	12 (8.3)	48 (5.8)	0.242	7 (2.8)	26 (3.2)	0.748

Values are means ± standard deviation or n (%). HGS, handgrip strength; ASMI, Appendicular Skeletal Muscle Mass Index; BMI, body mass index; PBF, percentage body fat; WC, waist circumference; C-index, conicity index. ^a^ Sarcopenia: low ASMI (<7.0 kg/m^2^ for men and <5.4 kg/m^2^ for women) and low HGS (<28 kg for men and <18 kg for women). ^b^ Sarcopenic obesity: sarcopenia with either high BMI (≥25 kg/m^2^) or high WC (≥90 cm for men and ≥85 cm for women). Statistical significance: * *p* < 0.05 and ** *p* < 0.01.

**Table 4:** Logistic regression analysis of sarcopenia and obesity parameters and fall history according to sex.

Characteristic	Unadjusted Model			Fully Adjusted Model		
	Men	Women	Total	Men	Women	Total
Low HGS ^a^	1.86	0.95	1.25	1.57	0.84	1.11
	(1.27–2.75) *	(0.67–1.35)	(0.96–1.61)	(1.02–2.42) *	(0.58–1.22)	(0.84–1.46)
Low ASMI ^a^	0.98	1.32	1.02	0.94	1.41	1.12
	(0.69–1.39)	(0.97–1.79)	(0.82–1.28)	(0.65–1.36)	(1.03–1.93) *	(0.89–1.42)
High BMI ^b^	1.06	1.03	1.09	1.16	1.01	1.07
	(0.73–1.53)	(0.77–1.37)	(0.87–1.36)	(0.80–1.70)	(0.75–1.35)	(0.85–1.34)
High PBF ^b^	1.70	1.13	1.36	1.90	1.20	1.46
	(1.14–2.52) *	(0.83–1.54)	(1.07–1.73) *	(1.26–2.86) *	(0.87–1.65)	(1.14–1.88) *
High WC ^b^	1.44	1.12	1.33	1.54	1.07	1.25
	(1.01–2.05) *	(0.84–1.49)	(1.06–1.66) *	(1.07–2.21) *	(0.80–1.44)	(1.00–1.57)
High C-index ^b^	1.54	1.38	1.77	1.59	1.31	1.52
	(0.98–2.42)	(0.74–2.56)	(1.24–2.53) *	(1.00–2.53) *	(0.70–2.46)	(1.06–2.18) *
Sarcopenia ^c^	1.7	0.98	1.20	1.45	0.96	1.18
	(1.09–2.65) *	(0.57–1.67)	(0.85–1.67)	(0.89–2.37)	(0.55–1.65)	(0.83–1.68)
Sarcopenic obesity ^d^	1.48	0.87	1.07	1.30	0.81	1.02
	(0.77–2.86)	(0.37–2.03)	(0.64–1.80)	(0.66–2.58)	(0.34–1.93)	(0.60–1.73)

Values are odds ratio (confidence interval). HGS, handgrip strength; ASMI, Appendicular Skeletal Muscle Mass Index; BMI, body mass index; PBF, percentage body fat; WC, waist circumference; C-index, conicity index. ^a^ Low HGS, <28 kg for men and <18 kg for women; low ASMI, <7.0 kg/m^2^ for men and <5.4 kg/m^2^ for women. ^b^ High BMI, ≥25 kg/m^2^; High PBF, ≥25% for men and ≥35% for women; high WC, ≥90 cm for men and ≥85 cm for women; high C-index, ≥1.25 m^3^/^2^·kg^1^/^2^ for men and ≥1.18 m^3^/^2^·kg^1^/^2^ for women. ^c^ Sarcopenia: low ASMI (<7.0 kg/m^2^ for men and <5.4 kg/m^2^ for women) and low HGS (<28 kg for men and <18 kg for women); ^d^ Sarcopenic obesity: sarcopenia with either high BMI (≥25 kg/m^2^) or high WC (≥90 cm for men and ≥85 cm for women); Statistical significance: * *p* < 0.05.

## Data Availability

All cohort data supporting the findings of this study are available from the KFACS and are open to all researchers upon reasonable request. All news articles published in the KFACS database, data provision manuals, and contact information are available on the KFACS website (http://www.kfacs.kr (accessed on 1 January 2020)).

## Abbreviations

The following abbreviations are used in this manuscript:

ASMI	Appendicular Skeletal Muscle Mass Index
AWGS	Asian Working Group for Sarcopenia
BMI	body mass index
CI	confidence interval
C-index	conicity index
DEXA	dual-energy X-ray absorptiometry
HGS	handgrip strength
KFACS	Korean Frailty and Aging Cohort Study
MMSE-KC	Mini-Mental State Examination—Korean version
OR	odds ratio
PBF	percentage body fat
WC	waist circumference
WHO	World Health Organization
